# Factors Associated With *Toxoplasma gondii* IgG and IgM Antibodies, and Placental Histopathological Changes Among Women With Spontaneous Abortion in Mwanza City, Tanzania

**DOI:** 10.24248/EAHRJ-D-16-00408

**Published:** 2017-07-01

**Authors:** Illuminata Machumi, Mariam M Mirambo, Deodatus Ruganuza, Peter Rambau, Anthony N Massinde, Albert Kihunrwa, Stephen E Mshana, Domenica Morona

**Affiliations:** a Department of Obstetrics and Gynaecology, Weill Bugando School of Medicine, Mwanza, Tanzania; b Department of Microbiology and Immunology, Weill Bugando School of Medicine, Mwanza, Tanzania; c Department of Medical Parasitology and Entomology, Weill Bugando School of Medicine, Mwanza, Tanzania; d Department of Pathology, Weill Bugando School of Medicine, Mwanza, Tanzania

## Abstract

**Background:**

*Toxoplasma gondii* infection in early pregnancy has been associated with significant adverse pregnancy outcomes. Despite being common in the city of Mwanza, its association with spontaneous abortion has never been studied. Here, we report the IgG and IgM seropositivity and histopathological changes of toxoplasmosis among women with spontaneous abortion.

**Methods:**

A total of 260 women with spontaneous abortion were enrolled between November 2015 and April 2016 from 4 hospitals in Mwanza city. Specific *T. gondii* IgG and IgM antibodies were detected from sera by indirect enzyme-linked immunosorbent assay (ELISA) while the conceptus tissues were stained with haematoxylin and eosin to demonstrate histo-pathological changes. Data were analysed by using Stata version 13.

**Results:**

The mean age of the enrolled women was 2665.9 years. The seropositivity of IgG and IgM antibodies were 144/260 (55.4%; 95% confidence interval [CI], 49–61) and 6/260 (2.3%; 95% CI, 3–8), respectively. IgG seropositivity was significantly high among women in the first trimester (59.1% vs.43.5%; *P*=.03). Only low gestation age (odds ratio [OR] 1.11; 95% CI, 1.02–1.20; *P*=.02) and keeping a cat (OR 11.80; 95% CI, 1.32–10.5; *P*=.03) independently predicted IgG and IgM seropositivity, respectively. Presence of inflammation (OR 1.95; 95% CI, 1.05–3.64; *P*=.03), calcification (OR 3.28; 95% CI, 1.01–10.63; *P*=.04), necrosis (OR 2.86; 95% CI, 1.39–5.89; *P*=.04), and lymphocyte infiltrations (OR 2.24; 95% CI, 1.17–4.24; *P*=.01) were significantly associated with *T. gondii* IgG seropositivity.

**Conclusion:**

Almost half of women with spontaneous abortion in the city of Mwanza have specific *T. gondii* IgG antibodies. Placental histopathological changes suggestive of toxoplasmosis were significantly found among IgG seropositive women. This calls for the need to screen these women during antenatal visits in order to institute appropriate measures, such as treatment and counselling, to prevent complications associated *T. gondii* infection.

## BACKGROUND

Toxoplasmosis is a common infection caused by a coccidian intracellular protozoan parasite, *Toxoplasma gondii*, which occurs in domestic animals and humans throughout the world. It is a public health concern by reason of its neurological manifestations among human immunodeficiency virus/acquired immunodeficiency syndrome (HIV/AIDS) patients and the potential association with adverse pregnancy outcomes.^1–4^ The infection is mainly acquired through ingestion of undercooked or raw meat containing viable cysts, ingestion of food and water contaminated with oocysts shed by cats, or congenitally during pregnancy.^5–7^ The course of the primary infection is often subclinical, with a majority of the infected individuals remaining asymptomatic; few patients may present mild symptoms.^8,9^ Primary infections during pregnancy are often asymptomatic but may result into foetal complications like spontaneous abortions, stillbirths, severe congenital malformations, and central nervous system symptoms in apparently normal infants.^10–13^ There is a geographical variation of the epidemiology of *T. gondii* infection. One-third of the world's population is estimated to be infected with *T. gondii*.^14,15^ About 0.01% to 0.1% of infants in developed countries are affected by congenital toxoplasmosis.^16^ Primary infection during the third trimester carries a higher risk of congenital infection than infection in the first and second trimesters.^16–18^ However, severe foetal sequels occur when the disease is acquired in the first trimester.^8,19^ Though data from African countries are still scarce, a few studies carried out in Tanzania have documented the magnitude of toxoplasmosis,^20–23^ however, none of these studies focused on women with spontaneous abortion. A high prevalence of toxoplasmosis among women with spontaneous abortions has been reported in Egypt and Mexico.^24,25^ Given the fact the *T. gondii* seropositivity among pregnant women is high in Mwanza^20^ there is a paramount need to investigate its association with poor pregnancy outcomes. The current study was undertaken in Mwanza to investigate the role of *T. gondii* infection as a potential cause of spontaneous abortion. The data collected may inform policy makers and prompt them to consider the need for a policy of screening and treatment of this infection during pregnancy to reduce possible associated complications.

## MATERIALS AND METHODS

### Study Design and Study Area

A cross-sectional hospital-based study was conducted between November 2015 and April 2016 involving 4 health facilities in the city of Mwanza, Tanzania. The 4 facilities included the Bugando Medical Centre (BMC), Sekou Toure regional hospital, Nyamagana district hospital, and Buzuruga health centre. These sites were purposively selected because they serve a large population of the city and provide obstetrics and gynaecological services for women with spontaneous abortion.

### Study Population and Inclusion and Exclusion Criteria

The study included all women diagnosed with spontaneous abortion in their first and second trimester of the pregnancy attending obstetrics and gynaecology clinics and emergency departments at the 4 selected sites. Women who were unsure about the dates of their last normal menstrual period and those in critical condition were excluded from the study.

### Sample Size Estimation and Sampling Techniques

The sample size was estimated by the Kish Leslie formula,^26^ using the prevalence of 12.8% from Kistiah et al.^27^ The minimum sample size calculated was 174, however a total of 260 women were enrolled. A serial sampling technique was used to enrol participants until the desired sample size was reached.

### Data Collection

Sociodemographic and medical/obstetric information was collected by a direct assessment of the study participants and pre-tested structured questionnaires.

### Sample Collection Procedure and Laboratory Investigations

During the collection of tissue samples, a checklist was provided to exclude observable signs of induced abortion, such as lacerations, cervical bruises, and foreign bodies during evacuation. A small sample of conceptus was collected and placed into 10% neutral buffered formalin for fixation. The tissues were subsequently processed and stained by haematoxylin and eosin as previously described.^28^ Slides were read by an experienced pathologist to detect the presence of necrosis, calcifications, plasma cells, and different forms of inflammation and to identify tachyzoites and bradyzoites.

For serological diagnosis, about 5 mL of venous blood was collected aseptically using plain vacutainer tubes (Becton, Dickinson and Company, Nairobi, Kenya). The samples were then taken to the Catholic University of Health and Allied Sciences (CUHAS) multipurpose laboratory where the serum was separated by centrifugation at 3,000 rpm for 5 minutes. The sera were kept at -40° C until processing. The detection of specific *Toxoplasma* IgM and IgG antibodies was done by commercial indirect enzyme-linked immunosorbent assay (ELISA) (PishtazTeb Diagno-stics, Teheran, Iran). The IgM ELISA assay used IgM capture principle. All procedures followed manufacturer instructions. A standard curve for IgG antibody detection was obtained by calibrating the standards 1 to 5 with concentration of 0, 10, 50, 100, and 200 IU/mL using ChemWell 2910 Automated EIA (Awareness Technology, Inc., Palm City, Florida, USA) as per manufacturer's instructions.

### Data Analysis

Data were entered into Microsoft Office Excel 2013. Coding and analysis were carried out using Stata version 13 (StataCorp, College Station, Texas, USA). Continuous variables, such as age, gestation age, gravidity, and antibody titres were summarized as median with interquartile range (IQR) or means with standard deviation. Categorical variables such as marital status, residence, education level, occupation, history of miscarriage, history of stillbirths, HIV status, keeping a cat, drinking unboiled water, or consuming pork, chicken, mutton, lamb, or beef were summarized as proportions. For the histopathological results, data were analysed using the Pearson's Chi-square test to observe the statistical differences of proportions in the various groups. In addition, the Wilcoxon Ranksum (Mann Whitney) test was used to compare differences on the medians between the groups. Univariate and multivariate logistic regression models were used to determine the predictors of *T. gondii* infection. Predictors with *P*-value of less than 0.2 were subjected into multivariate logistic regression analysis and their ORs and 95% CIs were noted. Predictors with *P*-values of less than 0.05 were considered statistically significant.

#### Ethics approval and consent to participate

Ethical approval was obtained from the joint CUHAS/BMC research ethics and review committee with ethical clearance number CREC/103/2015. Written informed consent was obtained from each participant prior to enrolment in the study. For participants aged below 18 years, consent was given by the parents/guardians who accompanied them.

## RESULTS

### Sociodemographic Characteristics of the 260 Women Enrolled in the Study

The mean age of enrolled women was 26±65.9 years. The majority (198, 76.2%) were in the first trimester, and 160 (61.5%) resided in rural areas. A total of 147 (56.5%), 86 (33.1%), and 27 (10.4%) had either no formal/primary, secondary, or tertiary education, respectively. The median gestation age of the enrolled women was 11 (IQR 9–13) weeks. A total of 114 (43.8%) of the enrolled women were housewives, while 58 (19.2%) and 88 (33.8%) were peasants or employed, respectively. The majority of women 185 (71.1%) were either primipara—a woman who is giving birth for the first time—or had at least 1 previous birth, as shown in Table 1.

**TABLE 1.** Sociodemographic Characteristics of the 260 Women With Spontaneous Abortion Enrolled in the Study

Characteristics	n (%)
Age^*^	26±5.9
Gestation age^*^	11 (IQR 9–13)
First trimester	198 (76.2)
Second trimester	62 (23.8)
**Education**	
No formal/primary	147 (56.5)
Secondary	86 (33.1)
Tertiary	27 (10.4)
**Residence**	
Rural	160 (61.5)
Urban	100 (38.5)
**Occupation**	
Housewife	114 (43.8)
Peasant	58 (19.2)
Employed	88 (33.8)
**Parity** Nullipara/0 children	75 (28.9)
≥1 children	185 (71.1)

* Mean age and median gestation age of the study participants.

Abbreviation: IQR, interquartile range.

### Seropositivity of Specific *T. gondii* Antibodies Among Women with Spontaneous Abortion

The seropositivity of IgG antibodies was 144/260 (55.4%; 95% CI, 49–61). Of the 198 women who were in the first trimester, 117 (59.09%) were IgG seropositive compared to only 27/62 (43.5%) of those in second trimester (*P*=.03). Regarding IgM seropositivity, the seropositivity was 6/260 (2.3%; 95% CI, 3–8).

### Factors Associated with Specific *T. gondii* Antibodies Among Women with Spontaneous Abortion

**FIGURE 1.**
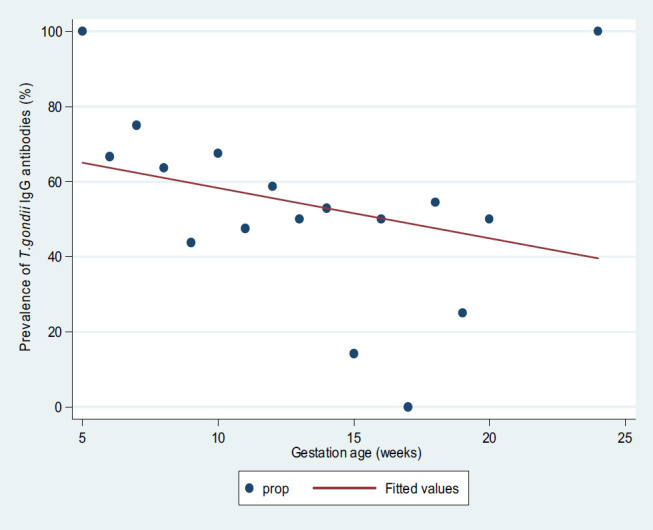
Graph of Prevalence of *T. gondii* IgG Antibodies by Gestational Age

The median gestation age of IgG seropositive women was significantly lower than the median gestation age of IgG sero-negative women (11 IQR 9–13 vs. 12 IQR 10–14; *P*=.02). On univariate logistic regression analysis, the decrease in gestation age (OR 1.09; 95% CI, 1.10–1.19; *P*<.03) was significantly associated with IgG seropositivity (Figure 1). There was no significant difference between the mean age of IgG seropositive women and that of IgG seronegative women (2665.9 vs. 2565.8; *P*=.17).

Out of 4 participants with HIV-positive status, 3 (75%) were IgG seropositive compared to 105 (55.3%) and 36 (54.6%) of those with HIV-negative status and those with unknown status, respectively (*P*>.05). Other factors investigated, such as residing in urban areas, history of previous miscarriage, history of stillbirth, drinking unboiled water, and keeping a cat were found to have a non-statistical increased risk of being IgG seropositive, as shown in Table 2. Only low gestation age (OR 1.11; 95% CI, 1.02–1.20; *P*=.02) was found to predict IgG seropositivity on multivariate logistic regression analysis (Table 2).

**TABLE 2.** Univariate and Multivariate Logistic Regression Analysis of Factors Associated With *T. gondii* IgG Seropositivity Among 260 Women With Spontaneous Abortion in Mwanza City

Characteristics (n)	IgG Seropositivity n (%)	Univariate OR (95% CI)	*P*–Value	Multivariate OR (95% CI)	*P*–Value
Age^*^	26±5.9	1.02 (0.98–1.07)	.188	1.03 (0.98–1.07)	.18
Gestation age^*^	11 (IQR 9–13)	1.09 (1.01–1.17)	.028	1.11 (1.02–1.2)	.02
Gravidity^*^	3 (IQR 1–4)	1.06 (0.92–1.22)	.397		
**Residence**					
Rural (160)	84 (52.5%)	1			
Urban (100)	60 (60%)	1.35 (0.81–2.25)	.237		
**Education**					
No formal/primary (147)	82 (55.8%)	1			
Secondary (86)	46 (53.5%)	1.00 (0.53–1.55)	.734		
Tertiary (27)	16 (59.2%)	1.15 (0.50–2.65)	.738		
**Occupation**					
Housewife (114)	57 (50%)	1			
Peasant (58)	37 (63.8%)	1.76 (0.92–3.37)	.087	1.54 (0.81–2.92)	.18
Employed (88)	50 (56.4%)	1.32 (0.75–2.30)	.336		
**Marital status**					
Single (38)	19 (50%)	1			
Married (222)	125 (56.3%)	1.23 (0.64–2.57)	.471		
**Previous miscarriage**					
No (221)	119 (53.9%)	1			
Yes (39)	25 (64.1%)	1.53 (0.75–3.09)	.237		
**HIV status**					
Negative (190)	105 (55.3%)	1			
Unknown (66)	36 (54.6%)	1.00 (0.55–1.77)	.920		
Positive (4)	3 (75%)	2.43 (0.24–23.77)	.446		
**History of stillbirth**					
No (225)	121 (53.8%)	1			
Yes (35)	23 (65.7%)	1.65 (0.78–3.47)	.189	1.51 (0.67–3.37)	.31
**Keeping a cat**					
No (170)	87 (51.2%)	1			
Yes (90)	57 (63.3%)	1.65 (0.97–2.78)	.062	1.29 (0.69–2.39)	.425
**Unboiled water**					
Yes (91)	56 (61.5%)	1			
No (169)	88 (52.1%)	1.47 (0.87–2.47)	.144		
**Beef**					
No (45)	25 (55.7%)	1			
Yes (215)	119 (55.4%)	0.99 (0.51–1.19)	.980		
**Chicken**					
No (84)	51 (60.7%)	1			
Yes (176)	93 (52.8%)	0.72 (0.42–1.23)	.233		
**Mutton**					
No (142)	85 (59.7%)	1			.235
Yes (118)	59 (50%)	0.67 (0.41–1.09)	.112	0.72 (0.43–1.23)	
**Pork**					
No (200)	105 (52.5%)	1			
Yes (60)	39 (65%)	1.68 (0.92–3.05)	.08	1.81 (0.95–3.44)	.06
**Lamb**					
No (215)	120 (55.8%)	1			
Yes (45)	24 (55.3%)	0.90 (0.47–1.72)	.761		

Abbreviations: CI, confidence interval; IgG, immunoglobulin G; IQR, interquartile range; OR, odds ratio.

Regarding the factors associated with IgM seropositivity, out of 169 women reported to keep cats, 5 (5.7%) were found to be IgM seropositive compared to only 1 out of 170 who reported not to keep cats (*P*=.037). Other factors such as history of stillbirth, drinking unboiled water, and not eating mutton or chicken were found to have a non-statistical increased risk of IgM seropositivity, as shown in Table 3. Only keeping cat (OR 11.8; 95%CI, 1.32–10.5; *P*=.03) was found to predict IgM seropositivity on multivariate logistic regression analysis (Table 3).

**TABLE 3.** Univariate and Multivariate Logistic Regression Analysis of Factors Associated With *T. gondii* IgM Seropositivity Among 260 Women With Spontaneous Abortion in Mwanza City

Characteristics (n)	IgG Seropositivity n (%)	Univariate OR (95% CI)	*P*–Value	Multivariate OR (95% CI)	*P*–Value
Age^*^	22±3.7	0.85 (0.72–1.02)	.083	0.88 (0.68–1.13)	.319
Gestation age^*^	12 (IQR 11–12)	0.99 (0.77–1.27)	.946		
Gravidity^*^	1 (IQR 1–2)	0.56 (0.26–1.19)	.134	0.74 (0.28–1.96)	.548
**Residence**					
Rural (160)	4 (2.5%)	1			
Urban (100)	2 (2%)	0.79 (0.14–4.42)	.794		
**Education**					
No formal/primary (147)	3 (2.04%)	1			
Secondary (86)	2 (2.3%)	1.14 (0.18–6.97)	.885		
Tertiary (27)	1 (2.3%)	1.84 (0.18–18.43)	.602		
**Occupation**					
Housewife (114)	4 (3.5%)	1			
Peasant (58)	1 (1.74%)	0.48 (0.052–4.41)	.519		
Employed (88)	1 (1.14%)	0.31 (0.034–2.87)	.307		
**Marital status**					
Single (38)	1 (2.6%)	1			
Married (222)	5 (2.2%)	0.85 (0.96–7.5)	.886		
**Previous miscarriage**					
No (221)	5 (2.3%)	1			
Yes (39)	1 (2.5%)	1.13 (0.12–10)	.908		
**History of stillbirth**					
No (225)	5 (2.2%)	1			
Yes (35)	1 (2.9%)	1.29 (0.14–11.41)	.816		
**Keeping a cat**					
No (170)	1 (0.5%)	1			
Yes (90)	5 (5.7%)	9.94 (1.14–86.44)	.037	11.8 (1.32–105.04)	.027
**Unboiled water**					
Yes (91)	3 (3.3%)	1			
No (169)	3 (1.8%)	1.88 (0.37–9.54)	.443		
**Chicken**					
Yes (176)	2 (1.14%)	1			
No (84)	4 (4.8%)	4.54 (0.31–10)	.093		
**Mutton**					
Yes (118)	2 (1.7%)	1			
No (142)	4 (2.8%)	4.54 (0.30–10)	.553		
**Pork**					
Yes (60)	1 (1.7%)	1			
No (200)	5 (2.5%)	1.52 (0.17–14)	.708		
**Lamb**					
Yes (45)	1 (2.2%)	1			
No (215)	5 (2.3%)	1.05 (0.11–10)	.967		
**Beef**					
Yes (215)	2 (0.93%)				
No (45)	4 (8.89%)	10.4 (1.8–58.8)	.008		

* Beef and chicken have collinearity with cat so they were not fitted on multivariate logistic analysis.

Abbreviations: CI, confidence interval; IgG, immunoglobulin G; IQR, interquartile range; OR, odds ratio.

### Histopathological Changes of Conceptus Products and *T. gondii* IgG Seropositivity

**FIGURE 2.**
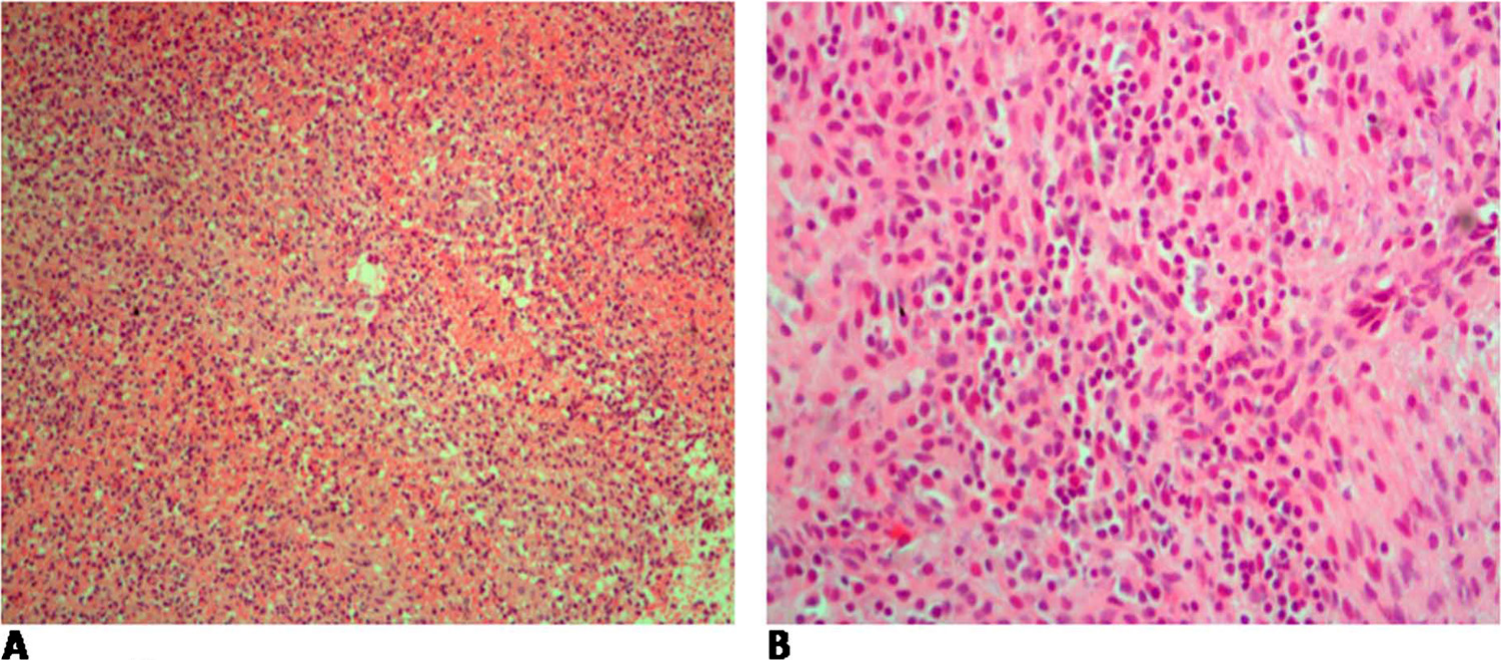
(A) Hematoxylin and Eosin Stain (10x), Neutrophils Infiltrates Signifying Acute Inflammation. (B) Hematoxylin and Eosin Stain (20x), Lymphocytes and Plasma Cells Signifying Chronic Inflammation.

**FIGURE 3.**
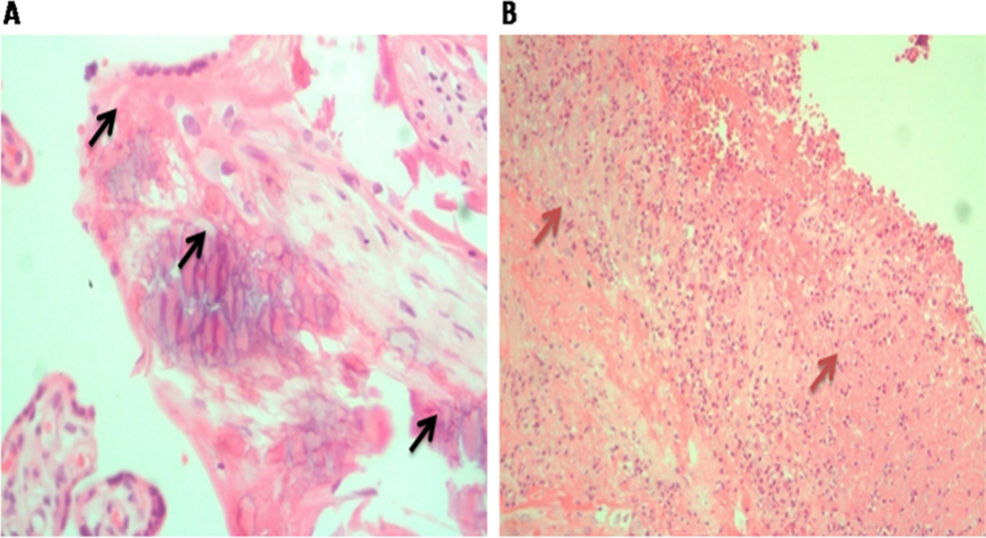
(A) Hematoxylin and Eosin Stain (20x), Black Arrows Showing Calcification. (B) Hematoxylin and Eosin Stain (10x) Brown Arrows Showing Areas of Necrosis with Neutrophils on the Background.

Conceptus products were obtained from 171 women. The histopathological changes examined included presence/absence of inflammation, including inflammatory cells such as macrophages, neutrophils, lymphocytes, and eosinophils, calcification, necrosis, and presence of tachyzoites and bradyzoites (Figures 2 and 3).

Presence of inflammation (OR 1.95; 95% CI, 1.05–3.64; *P*=.04), calcification (OR 3.28; 95% CI, 1.01–10.63; *P*=.05), necrosis (OR 2.86; 95% CI, 1.39–5.89; *P*=.01), and lymphocytes (OR 2.24; 95% CI, 1.17–4.24; *P*=.01) were significantly associated with specific *T. gondii* IgG seropositivity (Table 4). The presence of histopathological changes had a non-statistical association with increased IgG median titres.

**TABLE 4.** Histopathological Changes and IgG Seropositivity Among 171 Women With Spontaneous Abortion

Characteristics (n)	IgG Seropositivity	OR (95% CI)	*P*-Value
**Inflammation**			
Absent (103)	45 (43.7)	1	
Present (68)	41 (60.3)	1.95 (1.05–3.64)	.035
**Calcification**			
Absent (155)	74 (47.8)	1	
Present (16)	12 (75.0)	3.28 (1.01–10.63)	.047
**Necrosis**			
Absent (126)	55 (43.7)	1	
Present (45)	31 (68.9)	2.86 (1.39–5.89)	.004
**Macrophages**			
Absent (137)	64 (46.7)	1	
Present (34)	22 (64.7)	2.09 (0.95–4.56)	.064
**Lymphocyte**			
Absent (109)	47 (43.1)	1	
Present (62)	39 (63.0)	2.24 (1.17–4.24)	.014

Abbreviations: CI, confidence interval; IgG, immunoglobulin G; OR, odds ratio.

## DISCUSSION

*T. gondii* infections have been implicated in poor pregnancy outcomes and as a common cause of cerebral toxoplasmosis among patients infected with HIV. For the first time in Tanzania, we document a high seropositivity of specific *T. gondii* IgG antibodies among women with spontaneous abortion. Our findings are comparable to those reported elsewhere.^24,29^ In comparison to previous studies in Sudan and Tehran,^30,31^ the reported prevalence in the current study is indeed high. The difference could be attributed to a number of factors, such as geographical variation and climatic conditions, which have been found to influence toxoplasmosis worldwide.^32^ Moist and warm temperatures have been reported to enhance the sporulation of *T. gondii* oocysts. Generally, Mwanza's climate is warm with the temperature peaking around the third quarter of the year, which may explain high transmission rates. In addition, the prevalence reported in this study is significantly higher than what was reported in a previous study among pregnant women in the same setting and in an earlier report of data collected in the general population.^20,22^ The difference could be explained by the difference in study populations emphasizing the possible role *T. gondii* in causing spontaneous abortion.

The IgG seropositivity in this study was found to increase with an increase in maternal age, this confirms what was reported earlier.^20,33^ In addition, a lower gestation age was significantly associated with IgG seropositivity in this study. This corroborates the fact that most of the adverse *T. gondii* infection outcomes tend to occur during early pregnancy.^12^ Furthermore, women with a previous history of miscarriage and a history of stillbirth had increased odds of being IgG seropositivity, which was also reported previously.^31,34^

*T. gondii* IgM seropositivity in the current study is comparable to the previous study in Tehran, Iran, which reported a seropositivity of 2.7%.^31^ In contrast, the reported IgM seropositivityinthecurrentstudyislowcomparedtopreviousstudies in Sudan and Egypt.^24,29,30^ In comparison with a previous study conducted in the same settings^20^ among pregnant women with full-term delivery, the IgM seropositivity in the current study is indeed high. The presence of specific *T. gondii* IgM antibodies among these women may explain the primary infection, which is often associated with poor foetal outcome when acquired in the first 12 weeks of the pregnancy. However, in many cases, specific *T. gondii* IgM antibodies tend to persist longer after primary infection. Therefore, in these cases, an IgG avidity test is recommended to exclude the possibility of previous infections.^36^ Keeping cats was significantly associated with IgM seropositivity, which is consistent with other studies that reported the increased risk of *T. gondii* infections among pregnant women handling cats.^33,37^

This study observed that the odds of being IgG seropositive were significantly high among women with placental histo-pathological changes. It has been previously suggested that the mechanism by which *T. gondii* induce abortion is through achainofplacentalimmunologicalreactions.^24^ Animalmodel studies suggest that abortion can be induced by pathological changes, even without parasite replication in placental tissues.^38^ Inflammation was significantly associated with IgG seropositivity in the current study. This finding has also been reported in a previous study with a positive correlation betweenintensityofinflammationandpoorfoetaloutcome.^39^ Despite the fact that placental inflammation is non-specific and that the majority of incidents are of unknown origin, a positive correlation with IgG seropositivity suggests that *T. gondii* infection might have played a key role in this context.^40,41^ Highly sophisticated techniques are recommended to confirm the presence of parasites in placental tissues, since they can be easily overlooked in routine tissue studies. In addition, tissue necrosis and calcification have been found to be associated with IgG seropositivity in the current study, which is in agreement with a previous report.^42^ As a matter of fact, calcification and necrosis have been found to be common manifestations of toxoplasmosis.^43^ Generally, a positive association between IgG seropositivity and pathological changes, as observed in this study, suggests that *T. gondii* infection might have played an important role in spontaneous abortion in our setting. Further studies on the mechanisms through which *T. gondii* infection causes abortion are needed to provide a clearer understanding.

**TABLE 5.** Median Titres in Relation to Histopathological Changes Among 171 Women With Spontaneous Abortion

Characteristics (n)	Median IgG Titres (IU/mL)	IQR	*P*-Value
**Inflammation**			
Absent (45)	47.85	30.00–99.53	
Present (41)	75.93	30.00–202	.252
**Calcification**			
Absent (74)	54.22	27.19–154.38	
Present (12)	76.50	38.50–176.68	.278
**Necrosis**			
Absent (55)	52.22	30.00–118	
Present (31)	75.93	27.19–253	.244
**Macrophages**			
Absent (64)	54.22	30.54–118.18	
Present (22)	57.66	27.00–202.86	.922
**Lymphocyte**			
Absent (47)	52.22	30.00–99.77	
Present (39)	75.93	30.00–202.86	.312

Abbreviations: IgG, immunoglobulin G; IQR, interquartile range; IU, international units; mL millilitres.

### Limitations

The major limitations of this study were the failure to perform IgG avidity. Furthermore, sensitive techniques like polymerase chain reaction and immunofluorescent techniques for antigen detection could provide more information on the existence of parasite DNA in placental tissues.

## CONCLUSION AND RECOMMENDATIONS

The seropositivity of specific *T. gondii* IgG antibodies among women with spontaneous abortion and placental histopathological changes is alarmingly high in our setting, with a significant proportion of women at risk of contracting primary infection. The high prevalence of toxoplasmosis in women keeping cats, as one of the *T. gondii* IgM predictors, suggests that more education should be provided to these women on the risk of contracting *T. gondii* infection, especially during pregnancy. Ideally, these women should be excluded in activities that expose them to contact with infected cats or cat faeces, such as feeding cats and gardening. This study has provided baseline information on the association between *T. gondii* infection and spontaneous abortion in a particular setting. As toxoplasmosis is one of the TORCH infections—toxoplasmosis, other (syphilis, varicella-zoster, parvovirus B19), rubella, cytomegalovirus, and herpes infections—implicated in causing poor pregnancy outcomes, these findings may contribute to the improvement of antenatal care services and may trigger policy makers into considering screening and treatment for the women found with a *T. gondii* infection during antenatal visits. In addition, our results emphasize the need to consider *T. gondii* infection as one of the possible causes of spontaneous abortion. Overall, a better understanding of the infection and its outcome, and the implementation of the measures mentioned above may have a dramatic impact on the reduction of the adverse pregnancy outcomes associated with *T. gondii* infection.
